# High vagally mediated resting-state heart rate variability is associated with superior working memory function

**DOI:** 10.3389/fnins.2023.1119405

**Published:** 2023-02-20

**Authors:** Jia Zeng, Jiao Meng, Chen Wang, Wenwu Leng, Xiaoke Zhong, Anmin Gong, Shumin Bo, Changhao Jiang

**Affiliations:** ^1^The Center of Neuroscience and Sports, Capital University of Physical Education and Sports, Beijing, China; ^2^School of Information Engineering, Engineering University of People’s Armed Police, Xi’an, China; ^3^School of Kinesiology and Health, Capital University of Physical Education and Sports, Beijing, China

**Keywords:** vagal tone, HRV, working memory, rMSSD, fNIRS

## Abstract

**Background:**

Heart rate variability (HRV), a cardiac vagal tone indicator, has been proven to predict performance on some cognitive tasks that rely on the prefrontal cortex. However, the relationship between vagal tone and working memory remains understudied. This study explores the link between vagal tone and working memory function, combined with behavioral tasks and functional near-infrared spectroscopy (fNIRS).

**Methods:**

A total of 42 undergraduate students were tested for 5-min resting-state HRV to obtain the root mean square of successive differences (rMSSD) data, and then divided into high and low vagal tone groups according to the median of rMSSD data. The two groups underwent the n-back test, and fNIRS was used to measure the neural activity in the test state. ANOVA and the independent sample *t*-test were performed to compare group mean differences, and the Pearson correlation coefficient was used for correlation analysis.

**Results:**

The high vagal tone group had a shorter reaction time, higher accuracy, lower inverse efficiency score, and lower oxy-Hb concentration in the bilateral prefrontal cortex in the working memory tasks state. Furthermore, there were associations between behavioral performance, oxy-Hb concentration, and resting-state rMSSD.

**Conclusion:**

Our findings suggest that high vagally mediated resting-state HRV is associated with working memory performance. High vagal tone means a higher efficiency of neural resources, beneficial to presenting a better working memory function.

## 1. Introduction

Heart rate variability (HRV) represents the change in the time intervals between successive heartbeats (Wettstein et al., 2020). It is widely used as a safe, non-invasive, and reliable diagnostic tool to quantitatively analyze cardiovascular autonomic function (Zeki Al Hazzouri et al., 2018). HRV parameters can be obtained through time-domain, frequency-domain and non-linear analyses (Chan et al., 2015). On this basis, researchers can acquire some indicators underlying physiological mechanisms. In other words, while most HRV parameters evaluate the state of the parasympathetic nervous system (PNS), sympathetic nervous system (SNS), and other systems related to cardiac functioning, some specific HRV parameters can assess the contribution of PNS or SNS to cardiac function in a targeted way (Berntson et al., 1997; Laborde et al., 2017). Among them, the parameters that can measure the state of the PNS are indexed by the root mean square of the successive differences (rMSSD) in the time domain (Laborde et al., 2022). rMSSD mainly reflects parasympathetic activity mediated by the vagus nerve (Task Force of the European Society of Cardiology and the North American Society of Pacing and Electrophysiology, 1996; Lischke et al., 2019). The increase in rMSSD may mean an increase in vagal efferent drive (Shaffer and Ginsberg, 2017). Higher resting-state vagal tone can be regarded as a marker of psychological and physiological flexibility and has been proven to be related to some aspects relevant to psychophysiology phenomena. It also plays an important role in physical and mental health-related quality of life (Laborde et al., 2017).

In recent years, vagally mediated resting-state HRV has been gradually introduced into the study of working memory. Working memory refers to an ability to maintain and manipulate verbal and visuospatial information (Diamond, 2012). It is considered to be a complex but limited-capacity workspace that temporarily stores and processes information during cognitive tasks (Baddeley, 1992, 2001). As an important part of cognitive function, working memory plays an important role in learning, reasoning, problem-solving, and intellectual activities (Sumowski et al., 2010; Shipstead and Yonehiro, 2016; Chuderski and Jastrzębski, 2018; Yin et al., 2020; Yoo and Collins, 2022). Therefore, clarifying the relationship between the vagal nerve and working memory is of great significance. Some research suggests that working memory may be related to resting HRV.

However, it should be noted that their relationship remains somewhat controversial. Williams et al. (2019) reported no significant correlation between the test performance of subjects in the working memory task (Wechsler Adult Intelligence Scale, WAIS) and resting-state vagal tone. Stenfors et al. (2016) observed that higher standard deviation of normal to normal intervals (SDNN) and rMSSD were significantly related to improved performance in cognitive tests, including working memory. However, this relationship was no longer significant when using the age factor as a covariate. In addition, Hansen et al. (2003) found that the average reaction time and accuracy of soldiers in the high HRV group were better than those of soldiers in the low HRV group in the working memory test. Their results also revealed a significant association between vagal tone and working memory performance. Discrepancies among these results may be due to differences in working memory tasks, subject populations and age ranges. Through the literature review, it was found that previous studies mainly analyzed behavior performance related to working memory, and few experiments explored the latter from the perspective of brain function. Since many studies have shown that executive functions such as working memory are controlled by the prefrontal cortex (PFC) (Barbosa et al., 2020; Feng et al., 2021; Li et al., 2022), this study used fNIRS technology and combined it with the classic n-back working memory paradigm to explore the relations between vagal tone and working memory from the perspective of brain function and behavioral performance (Owen et al., 2005). fNIRS is a non-invasive neuroimaging technique with high temporal and spatial resolution. It can detect changes in cortical oxygenated hemoglobin (oxy-Hb) concentration in specific regions of the brain during executive tasks (Herold et al., 2018), and reflect differences in PFC activation when individuals complete the cognitive-behavioral tasks. At present, this technology has been widely used in cognitive neuroscience (Scholkmann et al., 2014; Pinti et al., 2020).

Based on this, this study intends to investigate the relationship between vagal tone and working memory, and combine the fNIRS to detect the oxy-Hb concentration of PFC-related brain regions of subjects with different vagal tone during the n-back task, to provide behavioral and cerebral hemodynamic evidence regarding the influence of vagal tone on working memory function. This study hypothesizes that high vagally mediated resting-state HRV is associated with behavioral performance and neural activity in the n-back task state. Specifically, subjects with higher vagal tone perform better on working memory tasks, and have lower oxy-Hb concentrations in the PFC during n-back tasks.

## 2. Materials and methods

### 2.1. Participants

Forty-two university students aged between 19 and 22 were recruited in this study. Their average weight was 67.66 ± 12.61 kg, and their average height was 175.12 ± 5.86 cm. All participants were right-handed. Exclusion criteria were: (1) neurologic or psychiatric disorders, (2) cardiovascular diseases, (3) current treatment with drugs. All participants provided informed written consent according to Ethical Committee guidelines.

### 2.2. Data acquisition

#### 2.2.1. Vagal tone

Both rMSSD and high frequency (HF) seem to reflect vagal tone (Shaffer et al., 2014). However, HF can reflect vagal tone only when it is between 0.15 Hz and 0.40 Hz, which implies a respiratory rate between 9 cycles per minute and up to 24 cycles per minute. Therefore, if the respiratory rate is outside this range, HF cannot accurately describe vagal tone. Compared with HF, rMSSD is relatively less affected by respiratory rate (Malik, 1996; Berntson et al., 1997; Hill et al., 2009; Shaffer and Ginsberg, 2017). Therefore, this study selected rMSSD as the indicator to assess vagal tone (Brown et al., 2013). HRV was measured as the rMSSD (Hansen et al., 2003; Afulani et al., 2021; Bellenger et al., 2021). Each RR wave Inter-Beat Interval (IBI) in the selected period was used to calculate rMSSD. IBIs were measured for 5 min using a Self-generate Physiological Coherence System (Heart Math-SPCS, Beijing HaoFeng), which received HR data from a sensor placed on the earlobe. The sampling rate was 1.3 Hz. All subjects were tested in quiet laboratory conditions with suitable light. The room temperature was controlled (22–24°C). In the 24 h before the test, the subjects did not take part in high-intensity physical activities or consume any alcohol, coffee, or drugs. Before the test started, subjects were asked to keep quiet, relax, and wear the equipment, and the testers debugged the equipment for testing.

#### 2.2.2. Working memory task

**FIGURE 1:**
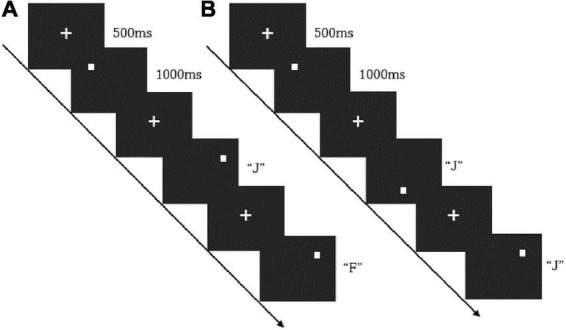
The flow path of the 1-back **(A)** and 2-back **(B)**.

The subjects’ working memory function was tested using the n-back task paradigm. The task consisted of sub-tasks of 1-back and 2-back difficulty, and the stimuli were spatial orientation pictures randomly presented on the screen. In the beginning, the target mark “+” appeared in the center of the screen, followed by the stimuli. The subjects needed to respond quickly to the stimuli with a maximum reaction time of 1,000 ms, and then the target mark “+” appeared again, and so on. In the 1-back task, the subjects needed to compare the position of the current picture with that of the previous one. If their position was the same, the subjects would press the “F,” if their position was different, they would press the “J.” The 2-back task required the subjects to compare the position of the current picture and that shown two trials earlier. They would press the “F” for the same position and the “J” for a different position. There is a 30-s rest between each block. The task flow is shown in Figure 1.

#### 2.2.3. fNIRS measurement

**FIGURE 2:**
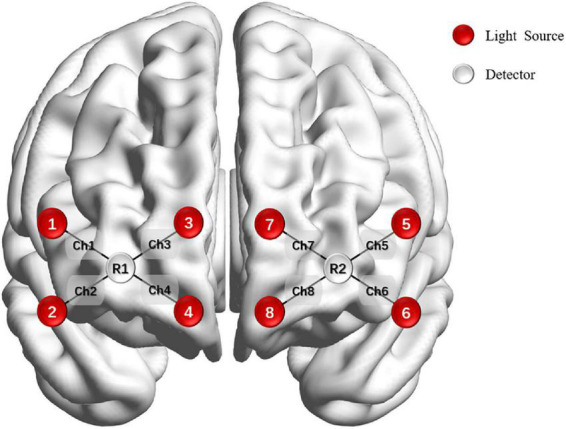
Optode placement on the forehead.

The OctaMon system (Artinis Medical, Netherlands) monitored PFC activity during the n-back test. Subjects sat on a chair in a quiet room, keeping their bodies and heads as still as possible. Brain oxygenation data were collected in the process of performing n-back tasks. The equipment had eight light sources and two detectors which allowed for eight channels distributed in the PFC, with 3.5 cm of source-detector separation. Optodes were positioned on the forehead using the international 10–20 system. The sampling frequency was 10 Hz (Figure 2).

### 2.3. Data processing and analysis

#### 2.3.1. Vagus nerve data

We imported ECG recordings into Kubios software (University of Eastern Finland, Finland), conducted a visual inspection of the full ECG recording, and manually corrected artifacts (Laborde et al., 2017). A medium filter was used (Papp et al., 2013; Pla et al., 2021). Then, rMSSD was calculated as the root mean square of successive differences of RR intervals (Colzato et al., 2018). Regarding HRV parameters, there were obvious abnormalities in the LF and HF indicators of two subjects, but their rMSSD data were normal. Since this study only used rMSSD data, no outliers were deleted. All participants were divided into the high HRV group (i.e., high rMSSD) and low HRV group (i.e., low rMSSD) according to their median rMSSD (Weber et al., 2010). This resulted in 21 subjects in each group.

Root mean square of successive differences data were determined to follow a normal distribution *via* the Kolmogorov–Smirnov test. Then, we used ANOVA to compare the differences in vagal tone between the high rMSSD group (HIGHs) and the low rMSSD group (LOWs). We set rMSSD as the dependent variable and took the groups (HIGHs and LOWs) as the independent variable, using age, gender, and body mass index (BMI) as the covariates. We used partial correlation controlling BMI, gender, and age to study the differences between the two groups. The ANOVA analysis used the Bonferroni correction.

#### 2.3.2. Behavioral data

Reaction time and accuracy are important evaluation indicators for n-back behavioral performance. E-Prime was used to collect and preprocess them. The outliers deleted from the reaction time data were determined as plus or minus three times the standard deviation.

In addition to reaction time and accuracy rate, behavioral performance was also measured by the inverse efficiency score (IES) (Lin et al., 2020). Reaction time is well known to be negatively associated with accuracy (Statsenko et al., 2020). Ignoring accuracy or analyzing reaction time and accuracy separately can affect the assessment of behavioral performance. Thus, IES, reaction time with consideration of accuracy, has been considered to be a good evaluation indicator (Hughes et al., 2014; Riedel et al., 2021). The formula is IES = RT/ACC (reaction time, RT; accuracy, ACC). IES and performance are negatively related, higher IES represents lower performance (Bruyer and Brysbaert, 2011). Afterward, differences between the high and low HRV groups on the measures of working memory performance were evaluated by the independent sample *t*-test.

#### 2.3.3. fNIRS data

Functional near-infrared spectroscopy data were preprocessed using Homer2 based on MATLAB (Mathworks, Natick, MA, USA). After the data was imported into Homer2, the raw light intensity signal was converted to optical density. We removed obvious bad segments and noisy channels first, and next made motion artifact correction. Then, we used a bandpass filter with a high-pass cut-off frequency of 0.01 Hz and a low-pass cut-off frequency of 0.1 Hz to eliminate the effects of cardiac oscillations, head movement, respiration and other factors. The optical density data were then converted into concentration changes of oxyhemoglobin and deoxyhemoglobin using the modified Beer–Lambert law. Finally, baseline correction was performed on the concentration data, and the average concentrations of oxy-Hb and deoxy-Hb in each channel under the task state of the subjects were calculated. Because oxy-Hb has a higher signal-to-noise ratio than deoxy-Hb, it is more sensitive to changes in cerebral blood flow (CBF) (Takeuchi et al., 2017), and some studies have pointed out that deoxy-Hb may reflect venous blood oxygenation and flow rather than local CBF characteristics. Therefore, oxy-Hb was used as the inspection index in the study (Takeuchi et al., 2016; Herold et al., 2021).

The oxy-Hb concentration data were normally distributed according to Kolmogorov–Smirnov tests. We used multivariate analysis of variance (MANOVA) analysis to compare the differences in oxy-Hb concentration between the two groups (HIGHs and LOWs). We set the oxy-Hb concentration of each channel as the dependent variable, with the groups (HIGHs and LOWs) as the independent variable. The MANOVA used Bonferroni correction.

Finally, to investigate whether vagal tone is related to behavioral performance and neural activities during n-back tests, our study used Pearson correlation coefficients to describe the potential relationships between vagal tone and working memory measures (both behavioral and oxy-Hb concentration correlates).

## 3. Results

### 3.1. rMSSD results

The median rMSSD of all subjects was 23.310 ms. The mean value of rMSSD was 25.740 ms (± 1.610) in HIGHs and 20.880 ms (± 1.533) in LOWs. Through the analysis of variance of resting rMSSD data, we found that the group effect was significant, and the rMSSD of HIGHs was significantly higher than that of LOWs [*F*(4,37) = 24.145, *p* = 0.000]. Therefore, it is believed that the resting-state vagal tone of HIGHs was found to be higher than that of LOWs. No significant effects were found for any of the covariates (age, gender, and BMI).

### 3.2. Behavioral results

**FIGURE 3:**
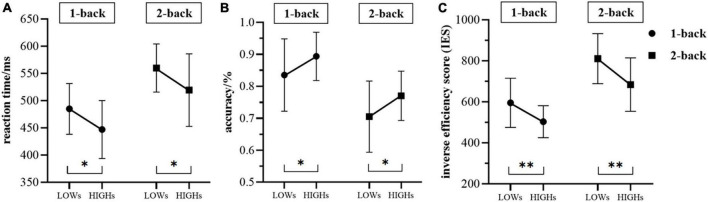
The differences in reaction time **(A)**, accuracy **(B)**, and the inverse efficiency score (IES) **(C)** between the two groups during the 1-back and 2-back tasks. “LOWs” and “HIGHs” refer to the test performance of subjects in the low rMSSD group and the high rMSSD group, respectively. Whiskers are standard deviation. LOWs, the low rMSSD group; HIGHs, the high rMSSD group. Asterisks indicate the level of statistical significance of *t*-tests. *p < 0.05, ^**^p < 0.01.

The reaction time of HIGHs in the 1-back task state was found to be significantly lower than that of LOWs (mean ± SD: HIGHs = 446.889 ± 53.174 ms vs. LOWs = 484.823 ± 46.603 ms, *t* = 2.459, *p* = 0.034), and the subjects in HIGHs also responded faster in the 2-back task state (HIGHs = 519.351 ± 66.629 ms vs. LOWs = 559.898 ± 44.166 ms, *t* = 2.324, *p* = 0.025) (Figure 3A). Furthermore, in the 1-back test, the accuracy of HIGHs was significantly higher than that of LOWs (HIGHs = 0.894 ± 0.056 ms vs. LOWs = 0.835 ± 0.113 ms, *t* = −2.126, *p* = 0.042). In comparison with the accuracy of the 2-back test, the accuracy of HIGHs was significantly better than that of LOWs (HIGHs = 0.770 ± 0.0773 ms vs. LOWs = 0.705 ± 0.111 ms, *t* = −2.199, *p* = 0.034) (Figure 3B). In terms of IES, the comparison results of each group were essentially consistent with the situation of the reaction time results. In both 1-back and 2-back conditions, the IES in LOWs was significantly higher than that in HIGHs (1-back: HIGHs = 502.966 ± 78.075 ms vs. LOWs = 594.847 ± 120.130 ms, *t* = 2.939, *p* = 0.006; 2-back: HIGHs = 683.988 ± 130.031 ms vs. LOWs = 810.371 ± 121.964 ms, *t* = 3.249, *p* = 0.002) (Figure 3C). Through the above comparison, it can be concluded that HIGHs had a shorter reaction time, lower IES, and higher accuracy in the n-back tasks and their overall performance was better than that of LOWs.

### 3.3. Oxy-Hb concentration

**FIGURE 4:**
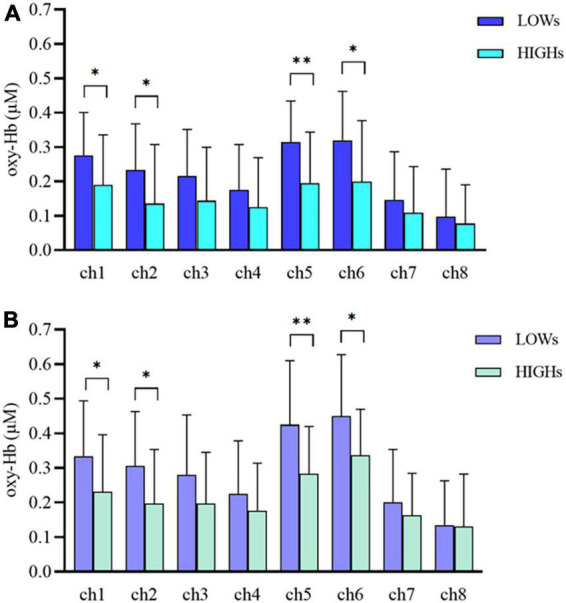
Oxygenated hemoglobin (Oxy-Hb) concentration changes in prefrontal cortex (PFC) during the 1-back **(A)** and 2-back **(B)** tasks within LOWs and HIGHs. The bar graph from ch1 to ch8 showing the mean oxy-Hb concentration value in each channel. LOWs, the low rMSSD group; HIGHs, the high rMSSD group; ch, channel. The horizontal bars represent means ± SD. *Bonferroni-adjusted *P* < 0.05 and ^**^Bonferroni-adjusted *P* < 0.01.

**FIGURE 5:**
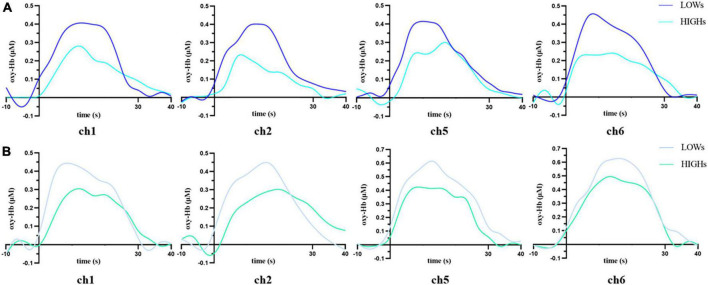
Time courses for concentrations of oxygenated hemoglobin (Oxy-Hb) in the prefrontal cortex of LOWs and HIGHs during the 1-back **(A)** and 2-back **(B)** tasks. The hemodynamic response of LOWs to n-back stimulation is higher than that of HIGHs. Stimulus block starts at 0 s and lasted for 30 s. −10 s to 0 s is baseline, and 30–40 s is post stimulus. ch, channel; LOWs, the low rMSSD group; HIGHs, the high rMSSD group.

We use MANOVA to compare the differences in oxy-Hb concentration between the two groups in the working memory tasks. Overall, the oxy-Hb concentration of the subjects in HIGHs when they were performing n-back tasks was lower than that of subjects in LOWs. Specifically, in the 1-back task, subjects in HIGHs had significantly lower oxy-Hb concentrations in channel 1 (ch1), ch2, ch5, and ch6 than those in LOWs [ch1: *H* = 0.190 ± 0.145 μM vs. *L* = 0.276 ± 0.125 μM, *F*(1,40) = 4.204, *p* = 0.047; ch2: HIGHs = 0.135 ± 0.172 μM vs. LOWs = 0.233 ± 0.134 μM, *F*(1,40) = 4.245, *p* = 0.046; ch5: HIGHs = 0.195 ± 0.149 μM vs. LOWs = 0.314 ± 0.119 μM, *F*(1,40) = 8.228, *p* = 0.007; ch6: HIGHs = 0.200 ± 0.177 μM vs. LOWs = 0.320 ± 0.142 μM, *F*(1,40) = 5.836, *p* = 0.020] (Figure 4A). By comparing the oxygenation data in the 2-back task, it was demonstrated that the oxy-Hb concentration of HIGHs in the channels ch1, ch2, ch5, and ch6 was significantly lower than that of LOWs [ch1: HIGHs = 0.231 ± 0.165 μM vs. LOWs = 0.334 ± 0.160 μM, *F*(1,40) = 4.222, *p* = 0.046; ch2: HIGHs = 0.198 ± 0.156 μM vs. LOWs = 0.305 ± 0.158 μM, *F*(1,40) = 4.950, *p* = 0.032; ch5: HIGHs = 0.283 ± 0.137 μM vs. LOWs = 0.425 ± 0.185 μM, *F*(1,40) = 8.004, *p* = 0.007; ch6: HIGHs = 0.336 ± 0.133 μM vs. LOWs = 0.450 ± 0.177 μM, *F*(1,40) = 5.571, *p* = 0.023] (Figure 4B). Grand averaged waveforms of statistically significant HbO concentration changes in the aforementioned channels are shown in Figure 5. This analysis showed that HIGHs had lower oxy-Hb in the PFC in the working memory task tests of different difficulty levels.

### 3.4. Correlations

**FIGURE 6:**
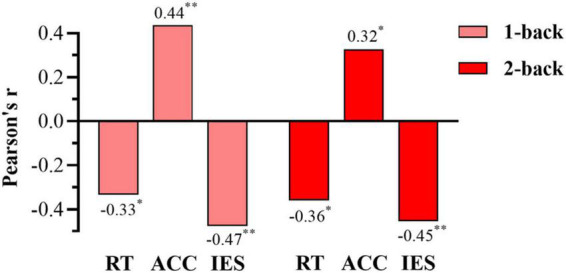
Bar chart of the correlations between root mean square of successive differences (rMSSD) and behavioral data in the 1-back and 2-back. RT, reaction time; ACC, accuracy; IES, the inverse efficiency score. *The correlation with *p*-value < 0.05, ^**^the correlation with *p*-value < 0.01.

We conducted Pearson correlation analysis on the behavioral data in the n-back tasks and rMSSD, and obtained the corresponding correlations between the behavioral data (reaction time, accuracy, and IES) of the 1-back and 2-back tests and rMSSD. Reaction time and IES in the 1-back test were significantly negatively correlated with rMSSD (RT: *r* = −0.334, *p* = 0.031; IES: *r* = −0.477, *p* = 0.001), and rMSSD was significantly positively correlated with accuracy (ACC: *r* = 0.436, *p* = 0.004). The same trend was found under the 2-back condition. Therefore, the reaction time, IES, and rMSSD were all significantly negatively correlated (RT: *r* = −0.360, *p* = 0.019; IES: *r* = −0.454, *p* = 0.003), and rMSSD was positively correlated with accuracy (ACC: *r* = 0.327, *p* = 0.035) (Figure 6). Overall, vagal tone represented by rMSSD was negatively correlated with the reaction time and IES of the n-back tasks and positively correlated with the accuracy.

**FIGURE 7:**
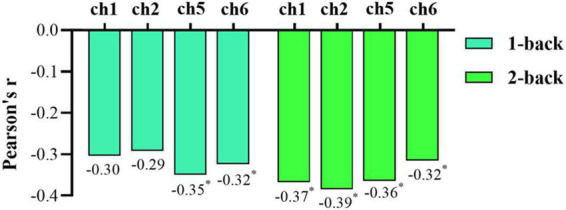
Bar chart of the correlations between root mean square of successive differences (rMSSD) and oxygenated hemoglobin (oxy-Hb) concentration during the 1-back and 2-back tasks. An asterisk (*) indicates statistical significance.

Pearson correlation analysis was also conducted to explore associations between the oxy-Hb concentration and rMSSD. In the 1-back test, ch1 and ch2 had a borderline significant correlation with rMSSD (ch1: *r* = −0.304, *p* = 0.050; ch2: *r* = −0.292, *p* = 0.061), and the oxy-Hb concentrations of ch5 and ch6 were significantly negatively correlated with rMSSD (ch5: *r* = −0.350, *p* = 0.023; ch6: *r* = −0.324, *p* = 0.036). For the 2-back task test, rMSSD was significantly negatively correlated with oxy-Hb concentrations in ch1, ch2, ch5, and ch6 (ch1: *r* = −0.368, *p* = 0.017; ch2: *r* = −0.385, *p* = 0.012; ch5: *r* = −0.364, *p* = 0.018; ch6: *r* = −0.315, *p* = 0.042) (Figure 7). Overall, rMSSD was negatively associated with the oxygenation level of brain region of interest (ROI) during the working memory task. In addition, compared with 1-back results, the oxy-Hb concentration of ROIs in the 2-back test was more strongly correlated with vagal tone.

## 4. Discussion

To our knowledge, this study may be the first to use fNIRS technology combined with behavioral tasks to investigate the relationship between vagal tone and working memory. By analyzing the behavioral and neuroimaging data of the two groups with different vagal tone, subjects with higher vagal tone were found to have significantly better reaction time, accuracy and IES in the working memory task test than those with low vagal tone. Moreover, the PFC of subjects with high vagal tone had lower oxy-Hb concentration when performing working memory tasks. There was a correlation between the resting-state vagal tone and working memory measures, including those related to reaction time, accuracy, IES and oxy-Hb concentration.

Behavioral data from this study suggested that subjects with high vagal tone have shorter reaction time, lower IES, and higher accuracy compared to those with low vagal tone, and such a difference in vagal tone can influence working memory performance. The correlation analysis revealed that there were significant relationships between resting-state rMSSD and behavioral performance. These results coincide with the outcomes of median split analyses using *t*-tests. Participants with high rMSSD showed more capability to match their working memory function to environmental demands. On the contrary, LOWs demonstrated the opposite characteristics of HIGHs. High rMSSD means higher vagal tone, which resulted in lower sympathetic stimulation so that the sympathetic nerve and the vagus nerve can reach a dynamic balance. When HRV increased, the function of the autonomic nervous system (ANS) was also enhanced, which improved the adaptability to the changes in the external environment. High HRV means good neuro-visceral integration in the body, a more flexible ANS, an improved ability to organize resources to meet demands, and the strengthened capability to quickly adapt to changing needs, thus improving behavioral control and cognitive performance. Hence, HIGHs performed better in all n-back tests than subjects with lower vagal tone in the behavioral study. The results were essentially in line with those of previous studies. Hansen et al. (2009) conducted cognitive tests on subjects with different vagal tones in different environments. The results showed that the high HRV group had a better accuracy independent of environmental demands. The reaction time of the low HRV group during threat-of-shock conditions was shorter, presumably because fear accelerates the information processing speed of the nervous system. The improved behavior of the high HRV group arises from the improved physiological and psychological regulation ability stemming from high vagal tone. Laborde et al. (2015) also found that young people with higher HRV performed better in tests using working memory tasks. Further hierarchical regression analysis showed a positive correlation between vagal tone and working memory performance. These two studies demonstrated that subjects characterized by increased vagally mediated cardiac control had improved working memory performance.

Functional near-infrared spectroscopy results demonstrated that subjects in the high HRV group had significantly lower oxy-Hb concentrations in the left and right PFC during working memory tasks compared to those in the low HRV group. This result was roughly consistent with that of the correlation analysis. Specifically, rMSSD was negatively correlated with oxy-Hb concentration in the prefrontal ROI of the subjects under the n-back condition. The higher the rMSSD, the lower the oxy-Hb concentration in the task-relevant brain regions was. However, we noticed that in the 1-back test, the oxy-Hb concentration of ch1 and ch2 had a borderline significant correlation with rMSSD. The reason may be that in the less difficult 1-back test, the brain regions corresponding to the left ch5 and ch6 were mainly involved in this task. Although the right ch1 and ch2 were also involved, the degree of ch1 and ch2 was lower than that of ch5 and ch6. The 2-back task is more difficult and requires higher hemoglobin levels than the 1-back task, so the oxy-Hb concentration in ROIs shows a more significant correlation with vagal tone in the 2-back task. Brain blood oxygen concentration influences working memory performance. Differences in the activation patterns of the related brain regions reflects differences in regional cerebral blood flow (rCBF) caused by the activation of neuronal cells. From the perspective of brain function, the PFC plays a vital role in executive functions such as working memory (Barbey et al., 2013; Shah et al., 2013). In this study, the high vagal tone group had lower levels of oxy-Hb concentration in the PFC while performing better in the task. Jansma et al. (2001) noted that it is of practical significance to interpret neural activity as a measure of task performance. Whereas decreased neural activity can reflect worse performance if a task is not sensitive to practice effects, it can reflect better performance if a task is sensitive to practice effects. Two previous studies using fNIRS reported decreases in PFC activation, with coinciding improvements in prefrontal-related cognitive performance (Hosseini et al., 2016; Moriarty et al., 2019). The decrease in task-related PFC activities and the improvement of behavioral performance of the high vagal group are possibly due to more efficient information processing in the neural network. A more efficient recruitment of neural resources may explain why subjects with high vagal tone can use fewer neural resources than those in the low vagal tone group when performing the same task (Jansma et al., 2001).

According to the neurovisceral integration model theory, PFC and limbic system (insula and cingulate gyrus) are connected with the hypothalamus and brainstem nuclei. These brainstem nuclei are influenced by cardiac vagal and sympathetic modulation (Thayer and Lane, 2000). This theory is supported by Mather’s research (Mather and Thayer, 2018), which pointed out that the slow oscillation in heart rate can enhance the functional connections of the PFC, cingulate gyrus, insula and other brain regions related to emotion, and cardiac activity can change related brain regions through humoral-neural pathways. Sensitivity to sensory input and high HRV mediated by high rMSSD improve cardiac function, which in turn modulates PFC activity. Using fMRI in both young and old at rest, Sakaki et al. (2016) found that higher HRV (measured using rMSSD) was relevant to higher functional connectivity in PFC and amygdala. In addition, Faes et al. (2017) also reported a brain-heart coupling relationship between the heartbeat dynamics signal and the gamma frequency band of the electroencephalogram (EEG) signal, and heart-to-brain information transfer is prevalently directed to the PFC during emotional elicitation.

In general, a higher vagal tone can desensitize the sympathetic nerve, slowing the speed and magnitude of sympathetic activation and thereby preventing sympathetic nerve overactivation after a particular stimulus. It can also promote the dynamic balance of sympathetic and vagus nerves, increase HRV, and enhance adaptability to changes in the external environment (He et al., 2015; Zhu et al., 2018). A high vagally mediated HRV implies a good degree of neurovisceral integration in the body, a more flexible ANS, a closer “brain-heart” coupling relationship, an improved ability to organize resources to meet needs, and the ability to quickly adapt to changing needs, thereby improving behavioral control and cognition performance (Porges, 2007; Deschodt-Arsac et al., 2020). This result was confirmed by previous studies of both young and old participants. Hansen et al. (2004) found that soldiers in the high HRV group performed better on working memory tests than those with lower HRV. Mahinrad et al. (2016) applied three interventions to three groups of elderly subjects: slow-rhythm breathing (6 bpm), normal rhythmic breathing (12 bpm), and spontaneous breathing, and revealed that subjects who performed slow-rhythm breathing at 6 bpm achieved synchronous oscillation, their HRV was significantly higher than that of the other two groups, and their working memory was positively correlated with HRV. Therefore, higher HRV can promotes the improvement of autonomic function through a dynamic balance of sympathetic and parasympathetic tension, thereby regulating the activity of related brain regions, decreasing PFC activation, and effectively improving working memory function.

## 5. Conclusion

This study combined behavioral tasks and fNIRS to examine the relationship between cardiac vagus nerve and working memory performance and prefrontal hemoglobin concentrations. Research has found an association between vagal tone and working memory. Higher vagal tone is conducive to promoting the dynamic balance between the sympathetic and vagus nerves, enhancing the ability of the ANS to regulate bodily functions. High vagal tone is associated with lower PFC oxygenation accompanied by a highly efficient use of neural resources, which are conducive to working memory function improvements.

## Data availability statement

The raw data supporting the conclusions of this article will be made available by the authors, without undue reservation.

## Ethics statement

The studies involving human participants were reviewed and approved by the Capital University of Physical Education and Sports Ethics Committee. The patients/participants provided their written informed consent to participate in this study.

## Author contributions

CJ, JZ, and JM designed the experiment. JZ, JM, WL, CW, and XZ were involved in data collection. JZ, AG, JM, CW, and XZ completed the data analysis. JZ and JM produced the first draft. CJ, AG, and SB checked the manuscript and gave advice about revising this manuscript. All authors read and approved the final manuscript.
